# Detection of the Candidate Genes of Economically Important Traits in Dorper Sheep Through Whole-Genome Resequencing

**DOI:** 10.3390/vetsci12090887

**Published:** 2025-09-14

**Authors:** Zhihua Wang, Zhengxi Liu, Hao Sun, Chunyan Bai, Te Pi, Huihai Ma, Zhongli Zhao, Shouqing Yan

**Affiliations:** 1 Department of Animal Science, Jilin University, Changchun 130062, China; zhihuaw24@mails.jlu.edu.cn (Z.W.); liuzhengxi@mail.kiz.ac.cn (Z.L.); sunhao92@jlu.edu.cn (H.S.); bcy@jlu.edu.cn (C.B.); pite24@mails.jlu.edu.cn (T.P.); 2 Institute of Animal Husbandry and Veterinary, Jilin Academy of Agricultural Sciences, Gongzhuling 136100, China; mahuih0819@163.com

**Keywords:** Dorper sheep, genetic diversity, growth, population structure, selection signature, whole-genome sequencing

## Abstract

Dorper sheep (DOR) are a commercially important mutton breed renowned for their high growth rate, favorable carcass composition, environmental adaptability, and natural wool shedding. In China, they are widely utilized as terminal sires to enhance growth and carcass yield in local breeds. To elucidate the genetic basis of these traits, we sequenced the genomes of 20 DOR and integrated the data with whole-genome sequences from 73 individuals representing four Chinese indigenous breeds. Analyses of genetic diversity, inbreeding coefficients, and population structure revealed reduced genomic diversity, elevated inbreeding levels, and clear genetic separation for DOR from other indigenous breeds. Selective sweep scans using *F*_ST_, *pi*, and XP-EHH identified candidate genes involved in five major trait categories: growth performance and development (*COL2A1*, *DAB2IP*, *EPYC*, *TSPAN18*, *WNT1*, *CTPS1*, *FBXW7*, *INSR*, *S100A6*, *SOCS2*), energy metabolism (*ACSS3*, *ADGRE3*, *CPT2*, *GCGR*, *PRKAA1*), fat deposition and adipocyte differentiation (*EHBP1*, *FOXP1*, *KLF12*, *PDGFD*, *RALGAPA2*), immune response (*CXCR6*, *IL17RB*, *NFKBIZ*, *TMEM154*), and wool traits (*CERS4*, *MITF*). These results will provide novel insights into the genomic architecture of economically important traits in DOR and support their genetic improvement through informed crossbreeding with Chinese local breeds.

## Simple Summary

Dorper sheep (DOR), developed in South Africa, are a globally important meat breed valued for their rapid growth, superior meat quality, adaptability to both hot and cold environments, and natural wool shedding, which reduces management costs. In China, DOR have been widely used as terminal sires in crossbreeding programs to improve growth performance and carcass traits of local breeds. However, the genetic mechanisms underlying these advantages remain largely unknown. In this study, we performed whole-genome resequencing of 20 DOR and compared them with four representative Chinese indigenous breeds. Our analyses revealed lower genomic diversity and higher inbreeding levels in DOR relative to local breeds, as well as distinct population structure separation. Selective sweep analysis identified candidate genes associated with growth performance and development, energy metabolism, fat deposition and adipocyte differentiation, immune response, and wool traits. These findings provide new genomic insights into DOR characteristics and offer a genetic basis for their targeted improvement through crossbreeding strategies.

## 1. Introduction

Sheep, one of the most important livestock species, were domesticated about 10,000 years ago in the Fertile Crescent and have continued to provide wool, pelts, milk, and meat for humans to the present day [1]. Today, there are many divergent breeds adapted to a wide range of natural environments and production systems around the world [2]. Interest in specialized breeds for mutton and lamb production has increased over the past two decades due to growing demand for lamb and declining wool prices [3].

Dorper sheep (DOR), native to South Africa, were developed in the 1930s by crossing South African Black-headed Persian with Dorset Horn sheep, and are now recognized as a major commercial mutton breed [4]. Originally bred for meat production under harsh environmental conditions, DOR are characterized by high growth rates, heavy carcasses, good meat quality, strong resistance to both cold and hot climates, efficient utilization of coarse grass and shrubs, and ease of management due to natural wool shedding, which eliminates the need for annual shearing [4,5,6,7]. In recent decades, DOR have spread worldwide and are widely used as terminal sires for lamb production [8].

In China, sheep were mainly raised for wool spinning until the late 1990s, when the mutton industry began to expand significantly [9]. Although Chinese indigenous breeds generally possess strong local adaptability and high reproductive rates, they tend to have smaller body sizes and slower growth rates compared to specialized commercial mutton breeds [10]. Over the past two decades, DOR have been introduced into China and are widely used as terminal sires in crossbreeding programs with local breeds, producing high-quality lambs with improved growth rates and carcass yields [11,12]. In addition, several breeds have been developed by crossing DOR with local sheep, such as Small-tailed Han sheep (STH), Hu sheep (HUS) and Mongolian sheep (MGS) [13], respectively.

Previous studies using mitochondrial DNA, microsatellite markers, microarray analysis, and limited genome resequencing have investigated the population structure, genetic diversity, and phylogenetic relationships of DOR [14,15,16]. However, due to the limited number of genetic markers identified, the genomic diversity and genetic basis underlying the prominent characteristics of DOR remain unclear. More recently, genomic studies have begun to explore selection signatures underlying the unique traits of DOR. Whole-genome resequencing of DOR and HUS identified candidate genes related to reproduction, muscle development, and immunity, enriched in signaling pathways such as PI3K–Akt and MAPK [8]. SNP chip-based comparisons of DOR from different countries revealed distinct signatures of selection potentially associated with environmental adaptation [17]. In addition, a comparative transcriptome analysis of liver and muscle tissues from DOR and STH identified 2188 differentially expressed genes, including *TGFB1*, *TGFB3*, *FABP3*, and *LPL*, which are likely associated with growth and meat quality traits [18].

Despite these advances, most genomic studies of DOR remain limited because of focusing on pairwise breed comparisons or specific traits. To fully characterize genomic diversity and selection signatures, comparative studies incorporating DOR and Chinese indigenous breeds are essential. Among modern Chinese local breeds, most are phylogenetically related to MGS. Ujimqin sheep (UJM), Tan sheep (TAN), STH, and HUS are all Mongolian subtypes that have adapted to diverse ecological environments under long-term domestication and selection, and they exhibit strong adaptability [13,19,20,21]. Here, we performed whole-genome resequencing of DOR and evaluated their genetic diversity, linkage disequilibrium decay, population structure, and genetic differentiation in comparison with UJM, TAN, STH, and HUS. In addition, we scanned for recent selection signatures and prioritized candidate genes related to growth, metabolism, fat deposition, coat and wool traits, immunity, and reproduction. These findings will provide new molecular insights into the genetic basis of DOR and contribute to future breeding and conservation strategies.

## 2. Materials and Methods

### 2.1. Sample Collection and Sequencing

Blood of 20 individuals was sampled from the purebred DOR population of Sheep Farm of Jilin Academy of Agricultural Sciences (Gongzhuling, China). DNA was extracted for whole-genome resequencing using the EasyPure Blood Genomic DNA Kit (TransGen Biotech, Beijing, China). DNA concentration and purity were measured with a NanoDrop 2000 spectrophotometer (Thermo Fisher Scientific, Waltham, MA, USA). Paired-end sequencing libraries were prepared for each individual and sequenced on the Illumina NovaSeq 6000 platform (Illumina, San Diego, CA, USA) with 150 bp paired-end reads at Novogene Bioinformatics Institute (Beijing, China). Additionally, genomic data of 73 individuals from four sheep breeds (UJM, *n* = 18; TAN, *n* = 18; STH, *n* = 18; HUS, *n* = 20) were downloaded from the Sequence Read Archive (https://www.ncbi.nlm.nih.gov/sra/, accessed on 8 March 2025) and analyzed to investigate the genetic diversity, population structure, and selection signals of DOR compared with other sheep breeds.

### 2.2. Alignments and Variant Identification

Raw reads were filtered and trimmed using fastp (v0.20.1) (https://github.com/OpenGene/fastp, accessed on 13 July 2025) with default settings [22]. Clean reads were aligned against the *Ovis aries* reference genome (Oar_rambouillet_v1.0) using BWA-MEM (v0.7.13) (https://sourceforge.net/projects/bio-bwa/files, accessed on 13 July 2025) with default parameters [23]. BAM alignment files were sorted by coordinate using SAMtools (v1.19) (https://www.htslib.org, accessed on 13 July 2025) to prepare for indexing and downstream analysis [24]. The Picard tool (v1.115) (http://broadinstitute.github.io/picard, accessed on 13 July 2025) was used to marker potential duplicate reads (REMOVE_DUPLICATES fault). Single nucleotide polymorphisms (SNPs) were identified using the Genome Analysis Toolkit (GATK, v4.4.1) (https://gatk.broadinstitute.org, accessed on 13 July 2025) following the recommended best practice workflow. The analysis was restricted to autosomal biallelic SNPs, and low-quality variants were filtered with the “VariantFiltration” function using the following thresholds: “QD < 2.0 || FS > 60.0 || MQ < 40.0 || SOR > 3.0 || MQRankSum < −12.5 || ReadPosRankSum < −8.0” [25]. In addition, the SNPs were filtered using PLINK (v1.9) (https://www.cog-genomics.org/plink, accessed on 18 July 2025) with the following parameters: (1) minor allele frequency (MAF) > 0.03; (2) SNP missing rate < 0.05; (3) individual missing rate < 0.10 [26].

### 2.3. Population Structure Analyses

Pairwise genetic distances were calculated in PLINK and used to construct a neighbor-joining (NJ) tree in MEGA 11 (https://www.megasoftware.net, accessed on 18 July 2025), with visualization in iTOL (https://itol.embl.de, accessed on 18 July 2025) [27,28]. To reduce marker redundancy, SNPs in high linkage disequilibrium were removed in PLINK using “--indep-pairwise 50 25 0.2”. The pruned dataset was used for principal component analysis (PCA) in GCTA (v1.92.3) (https://yanglab.westlake.edu.cn/software/gcta, accessed on 18 July 2025) and for model-based ancestry inference using ADMIXTURE (v1.3) (https://dalexander.github.io/admixture, accessed on 18 July 2025) with K values from 2 to 5 [29,30]. PCA and ADMIXTURE results were visualized in R (v4.4.1).

### 2.4. Genetic Diversity Analyses

Genetic diversity was assessed in DOR and four Chinese indigenous breeds (UJM, TAN, STH, HUS) using quality-filtered SNPs. The observed heterozygosity (*H*_O_) and expected heterozygosity (*H*_E_) were estimated in PLINK using the “--hardy” option. Nucleotide diversity (*pi)* was calculated in VCFtools (v0.1.16) (https://github.com/vcftools/vcftools, accessed on 18 July 2025) with the “--window-pi 50,000 --window-pi-step 25,000” [31]. Runs of homozygosity (ROH) were identified in PLINK using a sliding window of 50 SNPs, allowing at most one heterozygous genotype and up to two missing calls per window [32]. ROHs were defined as segments at least 300 kb in length, containing at least 58 SNPs, with a marker density of at least one SNP per 50 kb and no more than 100 kb between consecutive SNPs [33]. The genomic inbreeding coefficient (*F*_ROH_) was calculated as the proportion of the autosomal genome encompassed by ROHs for each individual. The inbreeding coefficient based on homozygosity (*F*_HOM_) was estimated in PLINK using the “--het” function. Linkage disequilibrium (LD) decay was assessed using PopLDdecay (v3.42) (https://github.com/BGI-shenzhen/PopLDdecay, accessed on 18 July 2025) by computing the squared correlation coefficient (r^2^) between pairwise SNPs across the genome with default settings [34].

### 2.5. Selection Signatures and Functional Annotation

To detect genomic regions under selection, we compared DOR with four Chinese indigenous breeds (UJM, TAN, STH, and HUS). Genome-wide selection signatures were assessed using three complementary approaches: fixation index (*F*_ST_), nucleotide diversity (*pi*), and cross-population extended haplotype homozygosity (XP-EHH). *F*_ST_ and *pi* were calculated in VCFtools with the parameters “--fst-window-size 50,000 --fst-window-step 25,000” and “--window-pi 50,000 --window-pi-step 25,000” [1]. The *pi* value was calculated as -ln (*pi*_DOR_/*pi*_REF_) and higher values indicate reduced nucleotide diversity in DOR relative to the pooled reference. Haplotype phasing and genotype imputation were conducted using BEAGLE (v5.4) (https://faculty.washington.edu/browning/beagle, accessed on 10 August 2025), and the resulting phased genotypes were used to compute XP-EHH scores with Selscan (v1.3.0) (https://github.com/szpiech/selscan, accessed on 10 August 2025) by comparing DOR with other reference breeds. XP-EHH values were normalized using the “--norm” function (v1.3.0) in Selscan with a window size of 50 kb [35]. For each method, the top 5% of windows were selected, and genomic regions overlapping across all three methods were defined as putative selective sweep regions. Candidate regions were annotated using SnpEff (v5.1d) (https://pcingola.github.io/SnpEff, accessed on 10 August 2025) to predict the functional effects of variants [36]. To investigate the biological significance of the candidate genes, Gene Ontology (GO) and Kyoto Encyclopedia of Genes and Genomes (KEGG) enrichment analyses were conducted using the DAVID online tool (https://davidbioinformatics.nih.gov, accessed on 10 August 2025). Of the statistics reported by DAVID, the raw *p*-value was used, and pathways with *p* < 0.05 were considered significant [37,38]. Additionally, Quantitative Trait Loci (QTL) data were obtained from the Sheep QTLdb (Release 56, 24 April 2025) (https://www.animalgenome.org/QTLdb, accessed on 15 August 2025) to identify overlaps between trait-associated QTL and the detected candidate regions [39]. Furthermore, haplotype patterns in candidate regions were visualized using the R package ComplexHeatmap (v2.25.2) (https://bioconductor.org/packages/ComplexHeatmap, accessed on 14 August 2025) [40].

## 3. Results

### 3.1. Sequencing and SNP Identification

Totally, 703.20 Gb raw data were obtained by the Illumina 150 bp paired-end platform and the detailed information on sequencing data of DOR is shown in Supplementary Table S1. Individual genomes of 20 DOR were generated to an average of 10.61× depth each and aligned to the reference genome with an average alignment rate of 98.06%. Finally, 26,496,611 high-confidence SNPs were retained across 93 sheep after quality control filtering in PLINK (Supplementary Table S2).

### 3.2. Population Structure Analysis

**Figure 1:**
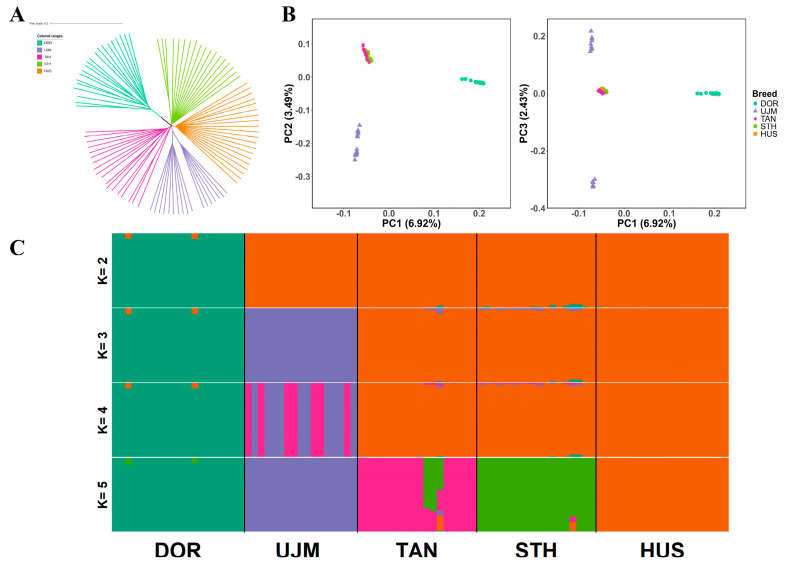
Population structure and relationships among DOR and four Chinese indigenous sheep breeds (93 individuals in total). (**A**) Neighbor-joining (NJ) tree showing genetic relationships among breeds. (**B**) Principal component analysis (PCA). Left: PC1 and PC2 (6.92% and 3.49% of variance explained, respectively); Right: PC1 and PC3 (6.92% and 2.43%). (**C**) Model-based clustering of individuals using ADMIXTURE with K = 2 to 5. Each vertical bar represents one individual, and colors represent the proportion of genetic ancestry assigned to each cluster. Abbreviations: DOR, Dorper sheep; UJM, Ujimqin sheep; TAN, Tan sheep; STH, Small-tailed Han sheep; HUS, Hu sheep.

The NJ phylogenetic tree revealed a clear separation of DOR from all four Chinese indigenous breeds (UJM, TAN, STH, and HUS), with the latter forming a closer genetic cluster (Figure 1A). After LD pruning, a total of 2,082,352 autosomal SNPs were retained for subsequent analyses. PCA further supported this pattern, with PC1 (6.92%) separating DOR from the Chinese indigenous breeds and PC2 (3.49%) further differentiating UJM from TAN, STH, and HUS (Figure 1B). Although PC1 and PC2 together explained ~10% of the total variance, the breed clustering remained consistent. Examination of PC1 versus PC3 (PC3 = 2.43%) did not reveal additional major differentiation, but we also observed that UJM individuals formed two subclusters, consistent with the NJ tree. Additionally, admixture analysis also confirmed the genetic distinctiveness of DOR; at K = 2, DOR formed an independent cluster, and at higher K values, the Chinese breeds exhibited additional substructure while DOR remained genetically distinct. When K = 5, all five breeds could be clearly distinguished (Figure 1C). Together, these analyses consistently demonstrated pronounced genetic divergence between DOR and Chinese indigenous sheep.

### 3.3. Genetic Diversity and Inbreeding

**Figure 2:**
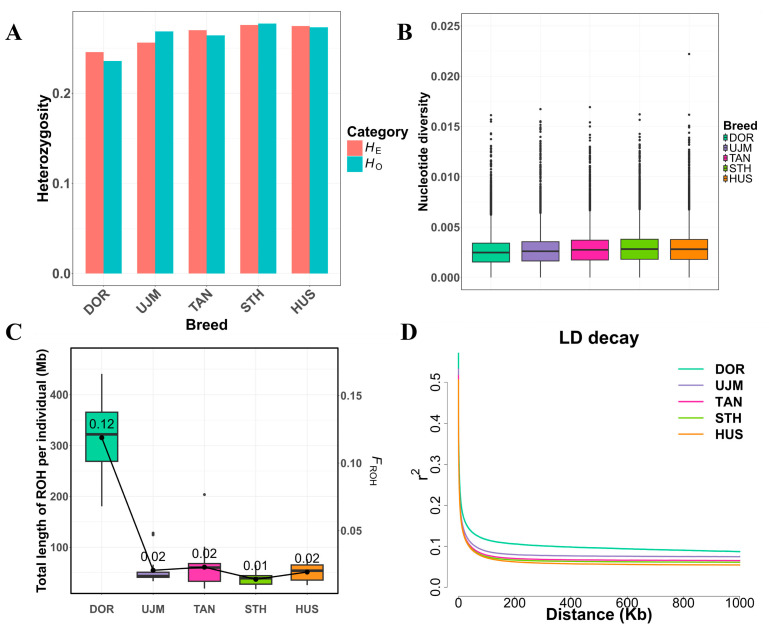
Summary statistics for genomic variation in 93 individuals from 5 breeds. (**A**) *H*_O_, *H*_E_ of each breed. (**B**) Box plots of *pi* for each breed. (**C**) Boxplot showing the total length of ROH per individual and the line plot showing the *F*_ROH_. (**D**) The decay of LD on sheep autosomes was estimated for each breed.

Across the five populations, DOR (*H*_O_ = 0.2357, *H*_E_ = 0.2456, *pi* = 0.0025) exhibited the lowest genetic diversity, while STH (*H*_O_ = 0.2773, *H*_E_ = 0.2757, *pi* = 0.0028) and HUS (*H*_O_ = 0.2732, *H*_E_ = 0.2746, *pi* = 0.0028) had the highest values. UJM (*H*_O_ = 0.2685, *H*_E_ = 0.2561, *pi* = 0.0026) and TAN (*H*_O_ = 0.2642, *H*_E_ = 0.2700, *pi* = 0.0027) showed intermediate levels. Overall, genetic diversity in the commercial DOR breed was lower than in all four Chinese indigenous breeds. (Figure 2A,B). Inbreeding estimated by the proportion of the genome in runs of homozygosity (*F*_ROH_) was highest in DOR (0.1188) and lowest in STH (0.0139). The homozygosity-based inbreeding coefficient (*F*_HOM_) showed a similar pattern, with DOR exhibiting the highest positive deviation from expected homozygosity (0.0401). The average ROH length per individual (KBAVG) in DOR was 0.5764 Mb, the highest among all breeds, followed by UJM (0.4437 Mb) and the shortest in STH (0.4178 Mb), suggesting the presence of longer homozygous segments likely resulting from stronger artificial selection and a reduced effective population size. Specifically for DOR, a total of 10,966 ROH fragments were detected, with lengths ranging from 0.3000 Mb on chromosome 17 to 3.2792 Mb on chromosome 11. The total ROH length per individual ranged from 180.6260 Mb to 440.5820 Mb, with an average of 315.5712 Mb, which was higher than those of the Chinese indigenous breeds (Figure 2C). For LD patterns, the fastest and slowest LD decay was observed in DOR and HUS, respectively (Figure 2D). It is consistent with the fact that DOR as a commercial mutton breed has undergone stronger artificial selection than local breeds. As expected, the pattern of LD decay was consistent with the result of the ROH profile in all five breeds (Supplementary Table S3).

### 3.4. Candidate Genomic Regions and Genes Under Selection

**Figure 3:**
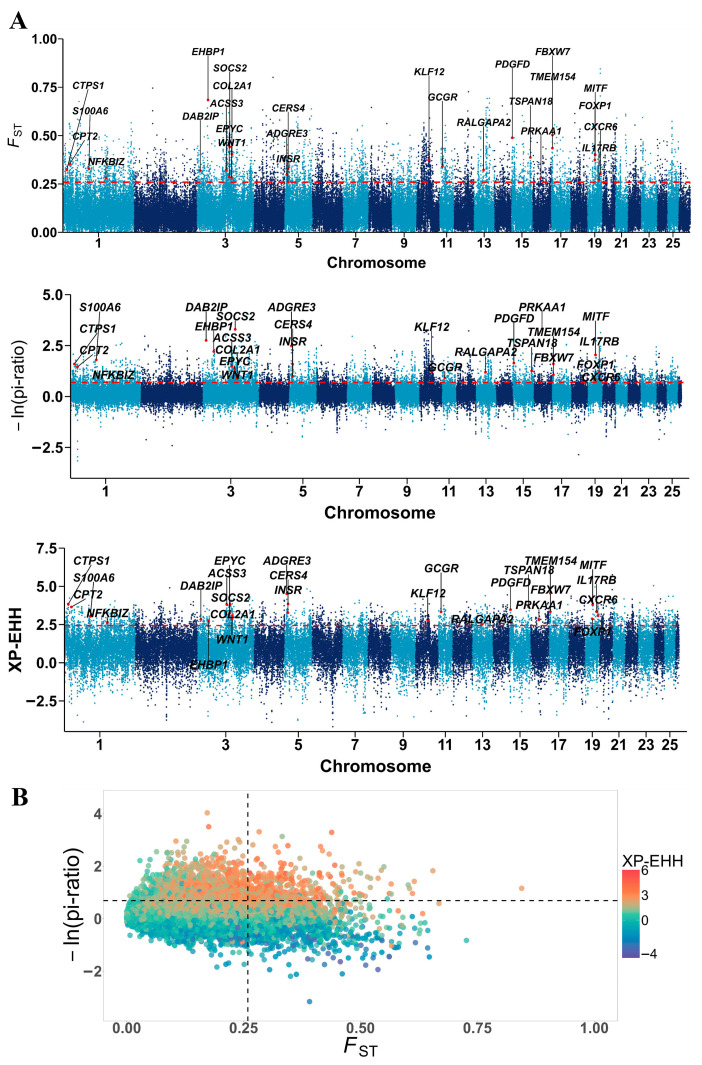
Analysis of the signatures of positive selection in the genome of DOR. Red dotted lines indicate the 5% significance threshold. (**A**) Manhattan plot of selective sweeps in DOR. (**B**) Conjoint analysis of the signatures of *F*_ST_, *pi*, and XP-EHH of DOR.

To detect genomic regions subjected to strong selection in DOR, we applied three complementary statistics: *F*_ST_, *pi*, and XP-EHH. The top 5% of *F*_ST_ windows contained 5294 regions encompassing 2981 genes, whereas *pi* identified 5141 regions harboring 2964 genes, and XP-EHH detected 2654 regions with 2048 genes. Intersection of the top 5% signals across all three methods revealed 1729 candidate regions spanning 50.25 Mb and annotated with 399 overlapping genes (Figure 3A,B). These regions likely represent footprints of directional selection associated with the breed formation and production traits of DOR (Supplementary Tables S4–S7).

Functional classification of these 399 genes, based on literature review and known trait associations, identified 26 strong candidates linked to economically important phenotypes (Table 1). Within growth performance and development, skeletal development genes included *COL2A1*, *DAB2IP*, *EPYC*, *TSPAN18*, and *WNT1*; muscle development genes comprised *INSR* and *SOCS2*; while *CTPS1*, *FBXW7*, and *S100A6* were implicated in cell growth regulation. In energy metabolism, candidate genes included *ACSS3*, *ADGRE3*, *CPT2*, *GCGR*, and *PRKAA1*. Fat deposition was represented by *PDGFD* and *RALGAPA2*, whereas adipocyte differentiation was associated with *EHBP1*, *FOXP1*, and *KLF12*. Coat and wool traits were linked to *CERS4* (hair follicle development) and *MITF* (pigmentation). Immune and disease resistance candidates included *CXCR6*, *IL17RB*, *NFKBIZ*, and *TMEM154*.

**Figure 4:**
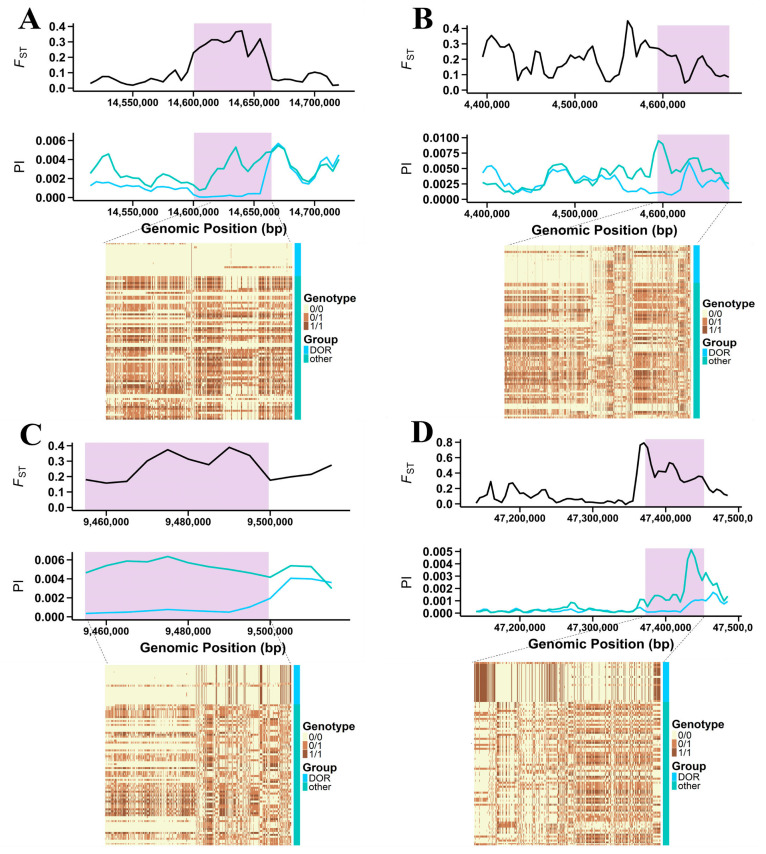
Haplotype and diversity patterns at four candidate genes under selection in DOR and other Chinese indigenous sheep. Each panel presents, from top to bottom, *F*_ST_, *pi*, and the haplotype structure of the two groups. The shaded purple regions mark the candidate windows: (**A**) *DAB2IP* (chr3: 14,600,468–14,664,440), (**B**) *PDGFD* (chr15: 4,593,970–4,675,672), (**C**) *ADGRE3* (chr5: 9,454,819–9,499,632), and (**D**) *EHBP1* (chr3: 47,371,974–47,452,647). In the *F*_ST_ and *pi* plots, the blue line represents DOR and the cyan line represents other sheep breeds. In the haplotype heatmap, homozygous reference genotypes (0/0) are shown in light yellow, heterozygous genotypes (0/1) in brown, and homozygous alternate genotypes (1/1) in dark brown. Abbreviations: DOR, Dorper sheep; other, four Chinese indigenous sheep (UJM, TAN, STH, and HUS).

After categorizing the 26 significant candidate genes into growth, lipid metabolism, immune response, coat traits, and energy metabolism, we further explored their biological relevance through GO and KEGG enrichment analyses of the full set of 399 intersecting genes. A total of 36 significantly enriched terms (*p* < 0.05) were identified (Supplementary Table S8), which were largely consistent with the trait categories inferred from gene annotation. Growth and skeletal development were highlighted by enrichment in terms such as skeletal system development (GO:0060348, *p* = 0.0210) and multicellular organism development (GO:0007275, *p* = 0.0371), involving *COL2A1*, *DAB2IP*, *EPYC*, *TSPAN18*, and *WNT1*. Cell growth regulation genes (*CTPS1*, *FBXW7*, *S100A6*) were associated with calcium signaling and protein phosphorylation pathways. Energy metabolism was supported by enrichment in calcium ion binding (GO:0005509, *p* = 0.0081) and ATP binding (GO:0005524, *p* = 0.0161), corresponding to *ACSS3*, *ADGRE3*, *CPT2*, *GCGR*, and *PRKAA1*. Immune and adaptation functions were reflected in inflammatory response (GO:0006954, *p* = 0.0010) and cell chemotaxis (GO:0060326, *p* < 0.01), linked to *CXCR6*, *IL17RB*, *NFKBIZ*, and *TMEM154*. In addition, analysis of the current sheep QTL database revealed that 40 QTLs were located within the candidate selective sweep regions, most of which were associated with growth, wool traits, immune response, and meat quality, including body weight, staple strength, staple length, fiber diameter, fecal oocyst count, fecal egg count, gastrointestinal nematode resistance, and water-holding capacity. These findings suggest that intensive selection for these economically important traits during DOR breeding has left a detectable genomic footprint (Supplementary Table S9). Furthermore, haplotype structure analysis was conducted for four key candidate genes (*DAB2IP*, *PDGFD*, *ADGRE3*, and *EHBP1*) located within selective sweep regions, all of which exhibited clear haplotype differentiation between DOR and the reference populations, consistent with their putative roles in growth performance, energy metabolism, fat deposition and adipocyte differentiation (Figure 4A–D).

## 4. Discussion

The characterizations of genetic diversity, population structure and selection signatures at the whole-genomic level are essential for genetic assessment, understanding germplasm characteristics, utilization and conservation of sheep genetic resources. In this study, the genomes of 20 DOR were sequenced, and the genetic diversity and selection signatures were analyzed in combination with 73 genomic data of DOR and four Chinese indigenous breeds available in the NCBI database.

We observed a lower level of genomic diversity in DOR than in the other four Chinese local breeds, suggesting that a substantial proportion of genomic variation has been lost during selection in the former but largely retained in the latter, consistent with the strong artificial selective pressure on DOR. Inbreeding estimates based on both *F*_ROH_ and *F*_HOM_ indicated elevated inbreeding in DOR, while most indigenous breeds showed values close to equilibrium between observed and expected homozygosity. DOR also exhibited the highest total ROH length, reflecting the presence of long homozygous segments likely resulting from intensive selection and reduced effective population size [33]. Notably, the pattern of LD decay was largely consistent with the ROH results across breeds.

Genetic selection has played an important role in improving productivity gains in animal breeding. In recent years, the identification of selection signatures in mammals has helped elucidate the mechanisms underlying many complex traits [67,68,69]. As a commercial mutton breed, DOR have been intensively selected for meat quality and production. To better understand the genetic basis underlying economically important traits, our study specifically focused on the genetic differentiation between DOR and other Chinese local breeds associated with growth performance and development, fat deposition and metabolism, coat and wool traits, immune and disease resistance, and reproductive performance. A total of 399 genes were identified by all three methods, which could be targets of recent or ongoing selection in DOR. We found that most of the strongest signals were in non-coding and intergenic regions, suggesting that these mutations could potentially be regulatory. A better annotation of the reference genome is therefore needed to identify the functional variants within these regions directly targeted by artificial selection. Some of these genes presented in our study have been reported to contribute to the genetic variation of these traits in some sheep breeds.

Growth performance and development in DOR were associated selection on genes involved in skeletal development, cell growth regulation, and muscle development. *COL2A1* maintains cartilage extracellular matrix integrity and supports endochondral bone growth; it was enriched in “protein binding” (GO:000551) [41]. *DAB2IP* has been linked to rib-number variation through regulatory networks with MESP1 and MESP2 [42]. EPYC contributes to cartilage matrix organization [43]. *TSPAN18* promotes endochondral ossification via VEGFR2-driven angiogenesis and Ca^2+^ signaling essential for osteoclast and osteoblast maturation, and was enriched in “inflammatory response” (GO:0006954) [44]. *WNT1* stimulates osteoblast differentiation and inhibits adipogenesis during bone development, enriched in “spinal cord association neuron differentiation” (GO:0021527), “bone development” (GO:0060348), and “negative regulation of cell–substrate adhesion” (GO:0010812) [45]. *CTPS1* functions in nucleotide biosynthesis and cell cycle regulation, potentially influencing tissue growth rates, with enrichment in “ATP binding” (GO:0005524) [48]. *FBXW7*, a substrate receptor of the SCF E3 ubiquitin ligase, controls G2–M transition by mediating degradation of key cell-cycle proteins [49]. S100A6 is a growth-responsive Ca^2+^-binding protein and the gene was enriched in “calcium ion binding” (GO:0005509) [50]. *INSR* activates PI3K–AKT and MAPK–ERK pathways to promote myofiber hypertrophy and proliferation, enriched in “multicellular organism development” (GO:0007275) [46]. *SOCS2* negatively regulates growth hormone signaling [47].

Energy metabolism-related genes regulate energy balance and substrate utilization. *ACSS3* deficiency in mice increases fat mass and induces insulin insensitivity via propionate accumulation in brown adipose tissue [51]. *ADGRE3* participates in acetate utilization to produce acetyl-CoA for lipid synthesis or energy; enriched in “calcium ion binding” (GO:0005509) [52]. *CPT2* plays a key role in fatty acid β-oxidation and energy production [53]. *GCGR* knockout mice show reduced adipose mass and resistance to diet-induced obesity through enhanced lipolysis and fatty acid oxidation [54]. *PRKAA1* acts as an energy sensor in ATP-deprived conditions, linking the AMPK–mTOR pathway to metabolic adaptation under hypoxia; enriched in “cellular response to calcium ion” (GO:0071277), “protein phosphorylation” (GO:0006468), “negative regulation of TORC1 signaling” (GO:1904262), and “Hypertrophic cardiomyopathy” (oas05410) [55]. Fat deposition and adipocyte differentiation-related genes are involved in adipocyte development and fat storage. *EHBP1* is essential for GLUT4 localization and insulin-regulated glucose transport in adipocytes, enriched in “protein binding” (GO:0005515) [56]. *FOXP1* represses brown/beige adipocyte differentiation and thermogenesis, with deficiency protecting against diet-induced obesity [57]. Reduced *KLF12* mRNA expression suppresses adipogenesis, accompanied by decreased expression of adipogenic transcription factors *(aP2*, *PPARγ*, and *C/EBPα)* [58]. *PDGFD* contributes to the fat-tail phenotype in sheep by promoting adipogenesis and maintaining adipocyte homeostasis [59]. *RALGAPA2* has been linked to subcutaneous fat thickness, feed intake, and body weight, indicating a role in fat deposition and carcass traits [60].

In addition, the ability for a sheep to shed its own wool seasonally can reduce the feeding cost and increase the economic income for farmers [70]. One of the reasons why DOR are among the most popular commercial mutton breeds is their characteristic shedding [70]. *CERS4* encodes ceramide synthase 4, which participates in producing key lipids for hair follicle structure [61]. *MITF* is a master regulator of pigmentation, with variants linked to light-colored wool formation [62]. *CXCR6* is essential for maintaining protective memory CD8^+^ T cells in the liver [63]. *IL17RB* is upregulated in paratuberculosis-affected sheep, suggesting a role in IL-25–mediated immune pathology [64]. *NFKBIZ* regulates inflammation and inflammation-related diseases via the NF-κB pathway [65]. *TMEM154* is associated with lentivirus susceptibility, and selecting resistant genotypes can help prevent infection [66].

In addition to these findings, several candidate genes identified in DOR have also been reported in other sheep populations, providing further context for our analysis. For example, *PDGFD*, *SOCS2*, and *RALGAPA2* were confirmed to be under selection in a large-scale comparative study of more than 30 breeds, including DOR and Suffolk sheep (SFK), consistent with their roles in adiposity, growth regulation, and carcass traits [1]. A recent review of meat-production traits in sheep also highlighted *INSR* and *SOCS2* in relation to growth and carcass yield, *CPT2* and *PRKAA1* in energy metabolism, and *PDGFD* as a central locus for adiposity and fat deposition [71]. Similarly, an investigation of Xinjiang sheep together with SFK and Dorset sheep identified *PDGFD* within the top selective regions associated with tail morphology and energy metabolism [72]. These concordant results across DOR and other international meat-type breeds suggest that part of the signals we observed represent common targets of selection, whereas other signals detected here may reflect characteristics shaped by the specific breeding history of DOR. The comparison of DOR with four Chinese indigenous breeds already provides a valuable framework for understanding recent selection, although further studies with larger sample sizes and additional breeds will be needed to refine these findings. Importantly, several of the genes identified in this study, including *PDGFD*, *INSR*, *SOCS2*, and *PRKAA1*, also represent promising molecular markers. Their polymorphisms could be incorporated into SNP arrays or PCR-based assays, offering clinicians and breeders practical tools to recognize and select for economically important traits.

## 5. Conclusions

DOR are a valuable and widely used commercial breed due to their rapid growth and superior meat quality. In this study, we provide a comprehensive analysis of the genetic diversity, population structure, and genomic differentiation signals of DOR compared with four Chinese native breeds. Our results revealed the relatively lower genomic diversity of DOR and identified several genes under past and ongoing selection that are associated with economically important traits, including growth and development, fat deposition and metabolism, and muscle development and meat quality. These findings provide a basis for further research on the genomic characteristics of DOR and for their development and utilization in crossbreeding programs with Chinese indigenous breeds.

## Figures and Tables

**Table 1:** Potentially selected genes associated with important economic traits in DOR were identified using *F*_ST_, *pi*, and XP-EHH analyses.

Chromosome	Position (bp)	Candidate Genes	Traits
3	148,725,001–148,775,000	*COL2A1*	Skeletal development [41]
3	14,600,001–14,650,000	*DAB2IP*	Skeletal development [42]
3	136,800,001–136,850,000	*EPYC*	Skeletal development [43]
15	80,850,001–80,900,000	*TSPAN18*	Skeletal development [44]
3	147,100,001–147,150,000	*WNT1*	Skeletal development [45]
5	14,475,001–14,525,000	*INSR*	Muscle development [46]
3	139,225,001–139,275,000	*SOCS2*	Muscle development [47]
1	16,125,001–16,175,000	*CTPS1*	Cell growth regulation [48]
17	6,000,001–6,050,000	*FBXW7*	Cell growth regulation [49]
1	110,075,001–110,125,000	*S100A6*	Cell growth regulation [50]
3	124,950,001–125,000,000	*ACSS3*	Energy metabolism [51]
5	9,475,001–9,525,000	*ADGRE3*	Energy metabolism [52]
1	29,350,001–29,400,000	*CPT2*	Energy metabolism [53]
11	12,050,001–12,100,000	*GCGR*	Energy metabolism [54]
16	35,450,001–35,500,000	*PRKAA1*	Energy metabolism [55]
3	47,325,001–47,375,000	*EHBP1*	Adipocyte differentiation [56]
19	31,900,001–31,950,000	*FOXP1*	Adipocyte differentiation [57]
10	52,800,001–52,850,000	*KLF12*	Adipocyte differentiation [58]
15	4,225,001–4,275,000	*PDGFD*	Fat deposition [59]
13	41,200,001–41,250,000	*RALGAPA2*	Fat deposition [60]
5	15,200,001–15,250,000	*CERS4*	Hair follicle development [61]
19	33,375,001–33,425,000	*MITF*	Pigmentation [62]
19	55,275,001–55,325,000	*CXCR6*	Immune response [63]
19	49,075,001–49,125,000	*IL17RB*	Immune response [64]
1	182,150,001–182,200,000	*NFKBIZ*	Immune response [65]
17	5,700,001–5,750,000	*TMEM154*	Immune response [66]

## Data Availability

The sequencing reads generated in this study have been deposited in the NCBI Sequence Read Archive (SRA) under the BioProject accession number PRJNA1130556.

## Supplementary Materials

The following supporting information can be downloaded at: https://www.mdpi.com/article/10.3390/vetsci12090887/s1, Supplementary Table S1: Summary of sequencing data; Supplementary Table S2: Summary of 73 sheep sample information; Supplementary Table S3: Genetic diversity of the 5 sheep breeds used in this study; Supplementary Table S4: A summary of genes from *F*_ST_ in DOR; Supplementary Table S5: A summary of genes from *pi* in DOR; Supplementary Table S6: A summary of genes from XP-EHH in DOR; Supplementary Table S7: A summary of genes overlapped by *F*_ST_, *pi* and XP-EHH methods in common regions; Supplementary Table S8: GO and KEGG enrichment analysis of DOR candidate genes by three methods; Supplementary Table S9: QTLs Overlapped with Candidate Selected Regions.
